# 6D Pose Estimation of Industrial Parts Based on Point Cloud Geometric Information Prediction for Robotic Grasping

**DOI:** 10.3390/e26121022

**Published:** 2024-11-26

**Authors:** Qinglei Zhang, Cuige Xue, Jiyun Qin, Jianguo Duan, Ying Zhou

**Affiliations:** 1 China Institute of FTZ Supply Chain, Shanghai Maritime University, Shanghai 201306, China; 2 Logistics Engineering College, Shanghai Maritime University, Shanghai 201306, China

**Keywords:** pose estimation, 3D point cloud, neural network, deep learning, appearance edge matching, robotic arm grasping

## Abstract

In industrial robotic arm gripping operations within disordered environments, the loss of physical information on the object’s surface is often caused by changes such as varying lighting conditions, weak surface textures, and sensor noise. This leads to inaccurate object detection and pose estimation information. A method for industrial object pose estimation using point cloud data is proposed to improve pose estimation accuracy. During the feature extraction process, both global and local information are captured by integrating the appearance features of RGB images with the geometric features of point clouds. Integrating semantic information with instance features effectively distinguishes instances of similar objects. The fusion of depth information and RGB color channels enriches spatial context and structure. A cross-entropy loss function is employed for multi-class target classification, and a discriminative loss function enables instance segmentation. A novel point cloud registration method is also introduced to address re-projection errors when mapping 3D keypoints to 2D planes. This method utilizes 3D geometric information, extracting edge features using point cloud curvature and normal vectors, and registers them with models to obtain accurate pose information. Experimental results demonstrate that the proposed method is effective and superior on the LineMod and YCB-Video datasets. Finally, objects are grasped by deploying a robotic arm on the grasping platform.

## 1. Introduction

Robotic grasping is one of the most fundamental yet challenging tasks in industrial automated manufacturing [1]. The goal is to replace humans in repetitive and tedious processes, such as part refilling [2], assembly, and sorting [3]. While most current approaches focus on pose estimation in scenes with scattered and occluded objects, few studies emphasize identifying the key objects necessary for operational tasks. In a typical robotic grasping scenario [4], the robot must recognize a target object within a cluttered environment, grasp it, and place it in a designated location [5]. A critical aspect of solving this problem is accurate attitude estimation [6], which involves determining the rigid transformation between the object’s coordinate system and the camera’s reference system—essentially, the six degrees of freedom for translation and rotation [7].

Recently, convolutional neural networks (CNNs) [8] have demonstrated outstanding performance in target detection, leading research in target recognition to increasingly focus on deep learning techniques [9]. Typically, feature extraction is performed using learning-based methods, with convolutional weights replacing traditional feature extraction to improve both efficiency and performance. For instance, PoseNet [10] employs a CNN architecture to predict 6D camera poses directly from RGB images. Current data-driven approaches aim to extract an initial pose from RGB images and refine it using algorithms like ICP [10] or MCN [11] algorithms. However, these methods tend to be computationally expensive and do not support end-to-end optimization. Newer strategies have emerged, incorporating visible appearance information from RGB images as auxiliary data for geometric inference in point cloud processing. These approaches place greater emphasis on using geometric information to enhance image representation learning. However, they tend to overlook the perception of the object’s pitch and roll in the point cloud data, while traditional 2D CNNs struggle with continuous geometric inference.

In contrast to RGB images, point clouds are an intuitive representation of the scene and are virtually independent of lighting conditions, helping to recognize textureless objects. The surfaces of industrial parts usually lack detailed surface texture. As a result, point clouds are used as a data format to efficiently and easily represent these untextured surfaces of industrial objects. In recent years, PointNet [12] and PointNet++ [13], as core architectures for processing point cloud data, have achieved remarkable results in point cloud categorization and semantic segmentation. This makes it feasible to derive features and determine the target pose from the point cloud. These methods better deal with the disordered and unorganized nature of point clouds and fully leverage the rich geometric details contained in point clouds, providing more effective solutions for target identification and pose estimation tasks. SGPN [14] performs instance segmentation directly on the point cloud, generating semantic information and instance representations for each point using a feature extraction model. In particular, SGPN divides semantics and instances into two separate branches. ASIS [15] adds information about each other for both branches to better achieve the performance of instance splitting. However, PointGroup [16] learns relative offsets through offset branches to move each point to the center of each instance to achieve simpler clustering performance. DenseFusion [7] fuses point cloud features extracted by PointNet and color features extracted by CNN at the dense pixel level. He et al. proposed the FFB6D [17] method for bitmap estimation, which performs representation learning by fusing geometric details from RGB appearance and point cloud and further enhances the representation of visual and geometric attributes with supplementary information provided by another branch.

Image segmentation primarily involves classifying the pixels in an image based on their categories [18]. When there are fewer pixels in a category, there will be an imbalance [19] between foreground and background categories. In addition, in multi-category datasets, when there are fewer types of objects in a single image, there will be a large number of invalid categories, meaning that most of the image consists of background categories. Therefore, the researchers proposed a large number of loss functions; the main loss functions include cross-entropy loss [20], balanced cross-entropy loss [21], and focal loss [22]. S. Kullback et al. [23] were the first to propose the cross-entropy loss function, and this loss function is used to measure the difference between two probability distributions. It demonstrates strong performance in the area of multi-class object image segmentation.

In addition, 3D keypoint descriptors were applied for pose estimation based on point cloud and depth information [24]. Alfred et al. [25] proposed a smoothing region growth algorithm for computing histogram descriptors of clustered viewpoint features for fast pose estimation. However, this method relies heavily on segmentation, leading to poor performance on industrial parts. To improve this, Drost et al. [26] proposed an advanced attitude estimation algorithm. The method combines voting and point-to-point features (PPF) to achieve a robust estimation of industrial part pose in complex and occluded scenes. Mohamad et al. [27] pointed out that the performance of the method degrades significantly when confronted with a large number of outliers or inaccurate normal estimates. Tuzel et al. [28] proposed a maximum margin learning architecture for recognizing key discriminative features of an object surface. These features can be ranked according to their importance in enhancing the precision of attitude estimation and reducing computational costs. Kiforenko et al. [29] evaluated the performance of PPFs, which shows that the four-dimensional descriptor PPF performs best in most datasets and has higher recall compared to other local histogram features. Jay et al. [30] proposed a 3D point cloud registration pose estimation network that combines image segmentation and real-time processing with convolutional neural networks.

In order to recognize and localize multiple types of objects in a cluttered scene, robots are able to grasp and manipulate objects accurately and reliably. This paper introduces a method for 6D pose estimation of industrial parts by robots through point-by-point instance segmentation and edge appearance matching. During the instance segmentation stage, the semantic category information of the target is obtained by incorporating a cross-entropy loss function. Accurate instance segmentation is achieved in the instance segmentation phase by means of semantic feature awareness of instance features. In the pose estimation phase, this paper proposes a PPF method based on the point cloud edge appearance model. This method can get the edge appearance keypoint features and use the point cloud normal features and deep gradient information for point cloud boundary point extraction, and finally, the reference point cloud and the instance point cloud after the instance segmentation for point cloud alignment to get the rotational and translational parameters of object position. The method uses point cloud edges for matching, which is faster than matching using the entire point cloud. To validate the method’s effectiveness, this paper evaluates the method on LineMOD [31], YCB-Video [10], and real datasets, respectively. The experimental results indicate that the proposed method surpasses current leading approaches.

The rest of the paper is organized as follows. Section 2 briefly outlines the logical structure of the paper. Section 3 evaluates the performance of the attitude estimation network and conducts robot grasping experiments; Section 4 then examines the experimental results and discussion, and Section 5 provides a summary of the study.

## 2. Pose Estimation Based on Geometric Information Prediction

**Figure 1:**
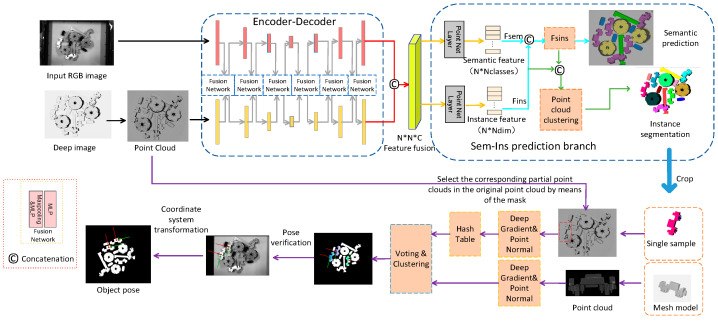
Posture estimation network structure. Feature extraction is performed on the given scene RGB and point cloud images, matching the target pose through semantic and instance prediction and designing a priority grasping strategy to achieve accurate grasping by the robotic arm.

Figure 1 gives the structure of the pose estimation method based on geometric information prediction, which uses RGB image and depth maps as inputs to detect a set of target objects in a set of scattered occlusion scenes and estimate their poses to achieve robotic grasping, which can greatly enhance the speed and precision of the robotic arm in gripping target workpieces in a disordered scene. The entire structure consists of three main modules. The first module introduces a feature extraction network where CNN is used to derive the appearance features from RGB images, and a point cloud network is used to derive geometric attributes from the point cloud data. In the feature extraction branches of both networks, a fusion module for the mutual mapping of 3D feature points to 2D feature points is added at each layer as a bidirectional communication scaffold for the fusion network. The two branches use local and global features from the other branch as auxiliary information to facilitate learning their own feature representations. The second module is the semantic-aware instance segmentation module, which uses the high-dimensional features generated in the first stage for subsequent semantic and instance feature prediction. The semantic category probabilities of different types of objects are obtained by employing the cross-entropy loss function. Inspired by the similarity metric, points of the same instance are near each other in the feature space, while the points from distinct instances remain separated in space. Based on this, the discriminant loss function is introduced to realize instance segmentation. The third module is the PPF pose estimation based on edge appearance, which involves two stages: offline data storage and online estimation. The key points at the edge of the point cloud are extracted by computing point cloud normal features and depth gradient information for each instance, and the first N key points are obtained by using the farthest point sampling method FPS [32], which calculates the point pair features, generates descriptors, registers the model point cloud with the instance cloud to obtain the initial pose, and finally calculates the rotational and translation parameters of the pose using the algorithm of ICP [10] to derive the target pose to obtain the grasping point pose. To ensure the robustness of the system against changes in the environment during grasping, the candidate poses are sorted and the top N poses are retained for grasping.

### 2.1. Bidirectional Dense Feature Extraction

The network structure takes as input data an aligned RGB image of a chaotically occluded view of the scene and the depth information converted from it. This stage forms the core of the network, where a CNN-based encoding-decoding branching network structure is used to derive the RGB image features, and RandLA-Net [33] is utilized for the point-cloud information to extract the global and local geometric features at a higher level of abstraction. Each point feature in the fused point-by-point dense features consists of a C-dimensional feature vector and is shared among all tasks the subsequent network performs.

The encoding and decoding process introduces a fusion network consisting of a shared perceptron MLP and maximum pooling. Given that the RGBD images are to be aligned, the network structure is linked by a 3D point cloud that connects pixel-by-pixel and point-by-point features. Given pixel i of a single RGB image, its coordinates in the 2D plane are ($x_{i},y_{i}$). By mapping the depth of each pixel i is mapped to its 3D point ($x_{i},y_{i},z_{i}$) using the camera’s internal reference matrix, and thus obtaining an XYZ map registered to the RGB image. The point feature $f_{pointj}$, which is aligned to the 3D point coordinates, is found to have its $K_{r2p}$ nearest neighbors on the XYZ graph, and the corresponding pixel points of these points and their appearance features are found from the appearance feature map and the appearance features of the nearest neighbors are aggregated by max-pooling and then passed through an MLP:

(1) $$f_{k_{r2p}}=MLP\left(\underset{1\leq i\leq k_{r2p}}{max}f_{i}\right)$$

where $f_{i}$ is the ith nearest neighbour in RGB and $f_{k_{r2p}}$ is the feature of the fused integrated pixel. Finally $f_{k_{r2p}}$ and $f_{pointj}$ are spliced using common MLP to generate the combined feature $M_{fusedpoint}$:

(2) $$M_{fusedpoint}=MLP\left(f_{pointj}⊕f_{k_{r2p}}\right)$$

where ⊕ is the concatenation operator.

In the same way, given point j in the point cloud, the 3D coordinate points in XYZ of each pixel are obtained based on the RGB map, and the nearest point of $K_{p2r}$ corresponding to it is found in the point cloud with its geometrical features, which are inputted into the multilayered MLP and maxpooling to map into a feature map with the same channel size as the RGB feature map:

(3) $$f_{k_{p2r}}=MLP\left(\underset{1\leq j\leq k_{p2r}}{max}f_{j}\right)$$

where $f_{j}$ is the jth nearest neighbour in the point cloud, and $f_{k_{p2r}}$ is the fused integrated point feature. Finally $f_{k_{p2r}}$ and $f_{rgb}$ are spliced using shared MLP to generate the combined feature $M_{fusedrgb}$:

(4) $$M_{fusedrgb}=MLP\left(f_{rgb}⊕f_{k_{p2r}}\right)$$

At each feature encoding and decoding layer, the feature mapping is scanned through a volume-as-kernel scan of the original feature mapping, which becomes smaller in height and width as the network goes deeper. To ensure that each feature pixel is mapped to its corresponding 3D coordinate, the average of the 3D coordinates is computed using a kernel of the same size, which generates the new 3D coordinates of the pixel point. However, the averaging operation may result in redundant noise due to the large depth variations of foreground targets and background boundaries in the environment. Therefore, the network uses a nearest neighbor interpolation algorithm to adjust the XYZ to a uniform size to match the feature map for feature fusion of subsequent appearance, corner point, and geometric information.

### 2.2. Object Detection Network

**Figure 2:**
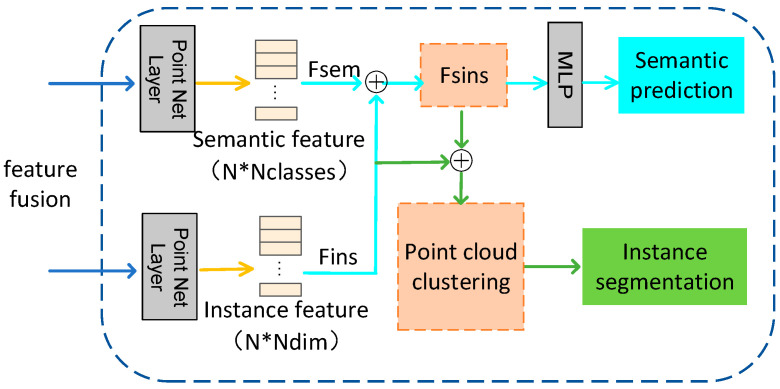
Instance segmentation branch network.

As shown in Figure 2, in the proposed target detection network in this study, pairs of dense appearance features and geometric features ($c_{i},c_{j}$) are extracted from aligned RGBD images and stitched together by a shared multilayer perceptual machine (MLP) to combine the complementary information of the two modalities effectively. The embedded point-by-point depth features $f_{i}\in R^{C}$ (each point contains C-dimensional features) are then fed into two separate branches to extract instance features and semantic features. Both branches utilize the PointNet network for learning, each focusing on a different feature extraction task.

In the instance feature branch, we employ a fully connected layer to generate a feature buffer in this study. This procedure aims to integrate local appearance information with global geometric structural information; therefore, the network’s capacity to identify object instances is enhanced. On this basis, the fused semantic features are further processed by a multilayer MLP, and the network assigns semantic labels to each point by learning the features of each point, thereby implementing semantic classification of point clouds. This paper also proposes an innovative feature fusion strategy. That is, fusing semantic features into instance features. Through this deep integration, instance features, when representing object pose and category information, can fully consider the impact of semantic information, thereby improving detection accuracy. Also, to further optimise the representation of instance features, we introduce a similarity-measure learning mechanism in this paper. It is centered on the idea that points in the same object instance should have similar characteristics; in contrast, the points in different object instances have significant differences. Consequently, Learning through similarity metrics, the network is able to ensure that points of the same object instance are close together in the instance feature space and different classes of points remain farther apart in the feature space. This effectively avoids confusion between categories. Therefore, different categories of points do not have the same distance in the instance feature space. That is, by semantically aware instance segmentation. In this paper, the semantic feature $F_{SEM}$ is further fused to instance feature $F_{INS}$. The formula is as follows:

(5) $$F_{SEM-INS}=F_{INS}+{wF}_{SEM}$$

where w is the category impact on instance features, where points belonging to different categories of instances are further excluded, while instances of the same category receive little impact. $F_{SEM-INS}$ is the instance features after fusing the semantic features and generating the final instance embeddings.

To ensure that there are significant differences between the instance characteristics of different objects in the category, a k-nearest neighbor (kNN) search is performed for each point in the embedding space of the generated instances in this paper. During the kNN search, we use marginal filtering to remove outliers and ensure that the K sampling points selected belong to the same instance. This marginal filtering technique works by embedding each point close to the mean value of the instance to ensure the stability of instance embedding while reducing the effect of noise on feature learning. In the instance embedding space, With kNN search, the semantic features of each point are organized into a feature tensor of shape $N_{P}\times K\times N_{f}$, where $N_{P}$ denotes the number of points in the instance, K is the number of neighborhood points returned by the kNN search, $N_{f}$ is the feature dimension of each point. Each set of features of this tensor represents a local region in the instance embedding space adjacent to the center of mass point of each instance. Specifically speaking, $N_{P}\times K$ is the index matrix produced by the kNN search and used to represent the relationship between each point and its K nearest neighbors. In order to extract the global semantic features of each instance, We used the channel-wise max aggregation (channel-wise max aggregation) method [34] to merge the semantic features of each group. This aggregation method selects the maximum of the semantic features of the K nearest neighbor points on each channel; thus, the features of each group are compressed into a representative feature vector. Specifically, for each instance center-of-mass point, Its semantic feature $y_{i}$ can be computed by the following equation:

(6) $$y_{i}=Max\left(x_{i1},\ldots ,x_{ik}\right)$$

where $\left(x_{i1},\ldots ,x_{ik}\right)$ denotes the semantic features of the K nearest neighbor points centered on point i. This maximal aggregation operation provides a fusion semantic feature for each center-of-mass point. After example fusion, The output of the feature tensor becomes a feature matrix $F_{INS-SEM}$ of shape $N_{P}\times N_{f}$. This matrix contains the global semantic features of each instance. Finally, a semantic classifier obtains the classification of different objects in the same instance to complete the point cloud instance segmentation.

### 2.3. Cross Entropy Loss Function and Discriminant Loss Function

The cross-entropy loss function is commonly used to handle classification tasks. It is used to describe the distance between two probability distributions. The smaller the cross-entropy value, the closer the two probability distributions are to each other. It is often used as a measure of the distance between predicted and actual values. In this paper, the use of common datasets for segmentation and recognition is framed as a multi-class classification problem. In other words, each sample belongs to only one category. The loss function for semantic branching is used by the classical cross-entropy loss [20] to train the network. It includes a softmax layer and a cross-entropy loss function.

(7) $$L=\frac{-1}{N}\sum_{i=1}^{N}\sum_{M=1}^{M}1\left\{y_{i}=M\right\}log\left({\hat{p}}_{i,M}\right)$$

where N represents the overall quantity of pixels in the scene image; ${\hat{p}}_{i,M}$ is the probability that pixel i belongs to category M as predicted by the model, and 1$\left\{y_{i}=M\right\}$ is the characteristic function, which takes the value of 1 when pixel i has a ground truth label of M and 0 otherwise.

For instance branches, since different classes of instance embeddings are learned independently, this means that semantic categorization and instance segmentation are not synchronized. Therefore, wrong semantic predictions may lead to errors in instance segmentation. Therefore, according to the discriminative loss function in [35], the variance term pulls the instance embeddings towards their average embeddings, while the distance term separates different objects in the same class to get multiple instance labels in the same class. The expression for this loss function:

(8) $$L=L_{var}+L_{dist}+L_{reg}$$

where $L_{var}$ is the average embedding that pulls the embedding toward the instances, while $L_{dist}$ keeps different instances of the same class separate, and $L_{reg}$ is a regularization that ensures that the instance embedding values are bound.

(9) $$L_{var}=\frac{1}{I}\sum_{i=1}^{I}\frac{1}{N_{i}}\sum_{j=1}^{N_{i}}{max\left({\left\|\mu_{i}-e_{j}\right\|}_{1}-\delta_{v},0\right)}^{2}$$

(10) $$L_{dist}=\frac{1}{I\left(I-1\right)}\sum_{i_{a}=1}^{I}{max\left({2\delta_{d}-\left\|\mu_{i}-e_{j}\right\|}_{1},0\right)}^{2}$$

(11) $$L_{reg}=\frac{1}{I}\sum_{i=1}^{I}{\left\|\mu_{i}\right\|}_{1}$$

where I is the count of instances within the label, $N_{i}$ represents the number of points in each instance, $e_{j}$ is the embedding representation of a point within an instance, $\mu_{i}$ is the average embedding per instance, $\delta_{d}$ and $\delta_{v}$ are the margins, and $max\left(x,0\right)$ denotes the hinge function.

In addition, to achieve instance segmentation, mean drift clustering [36] is used for each point, and the semantic labeling schema of points belonging to the same instance is used to determine their final category.

### 2.4. Pose Estimation Based on PPF for Point Cloud Edge Appearance and 4D Descriptors

After instance segmentation, we get the instance point cloud of the target object. Using object geometric information, such as depth gradients and point normals, a set of selected edge point pair features are computed to describe the target object in the example point cloud and the target model point cloud, respectively. Once the point-to-point features in the cloud of viewpoints are calculated if given a pair of scene points $\left(s_{r},{\;s}_{i}\right)$ and computing point-to-point feature descriptors, then using it as the key to find the corresponding model point pair feature ($m_{r}$,$\;m_{i}$) in the hash table. It is then possible to estimate the target’s pose by matching the scene’s point-to-point features with the model’s point-to-point feature set: offline computation of point-to-feature and online computation of point cloud alignment. Afterward, a scheme for point-to-feature voting in 2D space using local coordinates proposed by Drost et al. [26] was used. Calculating the correspondence between each ($m_{r}$,$\;m_{i}$) and $\left(s_{r},{\;s}_{i}\right)$, calculating the angle α between them, final voting in two dimensions, and recording the candidate poses corresponding to each scene point. The correspondence is then used to convert the model point cloud into a field point cloud to verify the correctness of the position. Finally, the ICP algorithm is used for positional refinement to achieve accurate object pose estimation information.

For industrial gripping scenarios, most of the time, there are low-textured, scattered stacks of parts. It is, therefore, necessary to fully utilize the geometric and local information of the visible parts of the object for pose estimation. The PPF method based on point cloud edge appearance is split into offline model data computation and real-time pose estimation. The offline processing phase consists of extracting the model point cloud’s boundary features using PCL techniques [37]. Boundary estimation, conversely, is based on the approach of Radu et al. [38], where boundary points are identified by analyzing the x, y, z gradients and normal directions of the points. Subsequently, the farthest point sampling (FPS) algorithm is performed on these key points, from which the top N key points are selected. Then, the features of all pairs of points for each key point are calculated, and features are generated and saved in the relevant hash table. As illustrated in

**Figure 3:**
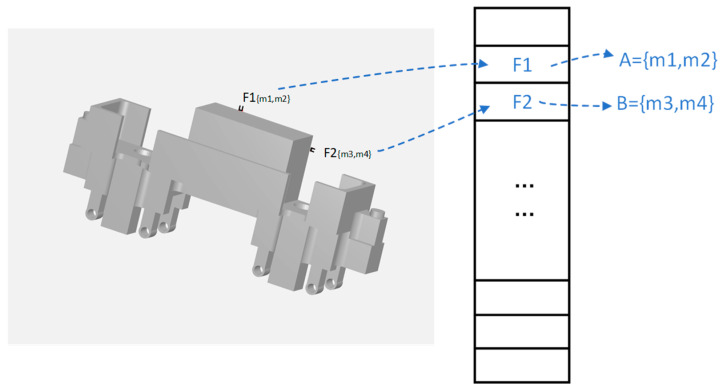
Hash table framework. For two point pairs in the model edge appearance, keypoint pair features are calculated and saved in the hash table for subsequent feature matching of the template point cloud with the instance point cloud.

Figure 3 The point pairs $(m_{1},m_{2}$) and $\left(m_{3},m_{4}\right)$ represent two point pairs in the point cloud while $F_{1}$ and $F_{2}$ are the point pair features computed through PPF.

The provided information consists of the point cloud data for a given scene; the pose information is dynamically computed for each instance. The bidirectional mapping between the depth map and point cloud is utilized to detect instance edge information. The curvature information of the point cloud is incorporated into the feature description when calculating the edge feature.

**Figure 4:**
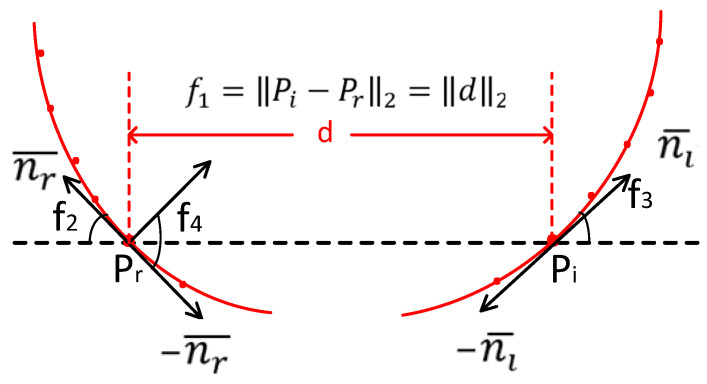
Definition of B2B-DL descriptors. In order to find corresponding pairs of points between the scene and the model, a descriptor was devised by calculating the tangent lines and considering their direction as the direction of the points.

The B2B-TL descriptor [39] is depicted in Figure 4. Revealing the corresponding point-to-point correspondence between the scene and the 3D model in order to calculate the target’s pose accurately. By employing these point pairs, a 6D pose can be calculated. Liu et al. [39] proposed that points located on the boundary of specific industrial components encompass more crucial information compared to those situated on a surface. The quantity of these points is significantly smaller than the number of points distributed across the object’s surface. One can opt to select boundary points to achieve attitude estimation in a shorter time. Typically, a multitude of corresponding point pairs can be found in the model and scene when utilizing the normal as the boundary point’s direction, and ensuring consistent selection of the same direction poses a challenge. Results in extended durations for aligning point clouds. The tangential direction of the point should, therefore, be chosen as its direction in order to enhance efficiency. The presence of tangents in two directions at each point can also result in inaccurate correspondences. The approach employed in this paper utilizes the direction of the tangent line, which forms an acute angle with the line connecting the two points, as the appropriate orientation to address this issue. Equation (12) computes the descriptor $F\in R^{4}$. $P_{r}$ and $P_{i}$ serve as the reference and reference points along the object’s perimeter, $\bar{n_{r}}$ and $-\bar{n_{r}}$ are the orientation of the point $P_{r}$, and $-\bar{n_{i}}$ and $\bar{n_{i}}$ are the directions of $P_{i}$.

The formula is as follows:

(12) $$F={\left(f_{1},f_{2},f_{3},f_{4}\right)}^{T}={\left({\left\|d\right\|}_{2},∠\left(\bar{n_{r}},d\right),∠\left(\bar{n_{i}},d\right),∠\left(\bar{n_{i}},\bar{n_{r}}\right)\right)}^{T}$$

where d denotes the vector extending from the reference point to the target point and $\left(V_{1},V_{2}\right)\in \left[0,\frac{\pi }{2}\right]$ denotes the angle between the two point vectors. $f_{1}={\left\|P_{r}-P_{i}\right\|}_{2}={\left\|d\right\|}_{2}$ indicates the Euclidean distance between two point clouds. $f_{2}$ is the acute angles between the vector d and the tangent line through the points $P_{r}$ and $f_{3}$ is the acute angles between the vector d and the tangent line through the points $P_{i}$. $f_{4}$ represents the acute angle formed by the two selected tangents. The method proposed by Drost et al. [26] was used with $d_{dist}$ and $d_{angle}$ as the step sampling distance and angle on the boundary.

Several initial pose candidates are generated using a voting scheme akin to that of Drost et al. [26], which sets up a local coordinate system based on points in 2D space.

**Figure 5:**
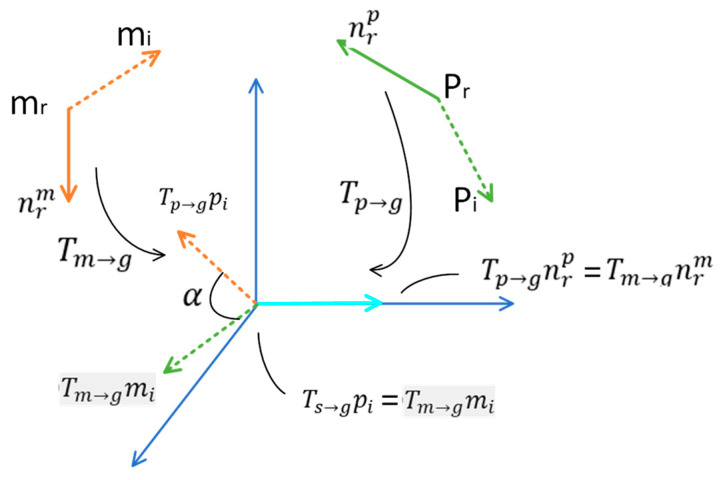
The coordinate transformation relationship between the instance and model point clouds. The inverse transformation $T_{p\to g}^{-1}$ repositions the reference point $p_{r}$ of the example point cloud to the origin, and the normal direction is aligned parallel to the x-axis of the coordinate system.

**Figure 6:**
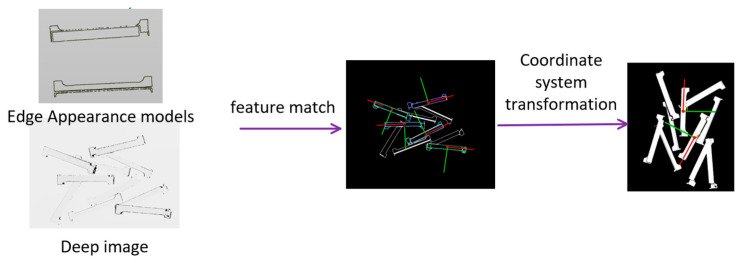
Positional information for each instance obtained by aligning the model point cloud with the field instance point cloud.

The normals $n_{p}^{r}$ and $n_{m}^{r}$ at each point are aligned with the x-axis, as shown in Figure 5. The reference points $p_{i}$ and $m_{i}$ are aligned along the x-axis. α and $m_{r}$ establishes the local coordinates ($m_{r}$, α). The reference point $m_{r}$ of the model point cloud, along with its normal direction, is determined by $T_{m\to g}^{-1}$. Align the reference point $T_{p\to g}^{-1}p_{i}$ of the transformed example point cloud with the reference point $T_{m\to g}^{-1}m_{i}$ of the transformed model point cloud, following the transformation of α around the x-axis Transform the transformed instance point cloud reference point $T_{p\to g}^{-1}p_{i}$ by an angle of α around the x-axis so that it is aligned with the transformed model point cloud reference point $T_{m\to g}^{-1}m_{i}$, and vote for ($m_{r}$,α). The ($p_{r}$,$p_{i}$) can be derived from Equation (13).

(13) $$p_{i}=T_{p\to g}^{-1}R_{x}\left(\alpha \right)T_{m\to g}m_{i}$$

where $T_{p\to g}^{-1}$ and $T_{m\to g}$ denote the transformations of the scene coordinate system and the model coordinate system to the intermediate coordinate system. $R_{x}\left(\alpha \right)$ represents a rotational transformation around the x-axis by an angle of α. Finally, voting is performed in the two-dimensional space ($m_{r}$,α). As shown in Figure 6, the alignment of the model point cloud with the field instance point cloud according to the coordinate transformation relationship can obtain the position information of each instance, and for the highest voted local coordinates, Equation (8) was used to calculate the transformation relationship between the model and the scene coordinate system.

Next, pose verification is performed following Li et al. [40] to eliminate incorrect candidate poses. The candidate poses obtained were refined utilizing the iterative closest point (ICP) algorithm [36]. Ultimately, the refined pose results are fed into the grasp planning, yielding the final gripping information.

### 2.5. Gripping Planning

After the pose estimation network gets the 6D position information of multiple instances of the target object, it generates a list of candidate positions, i.e., given a set of candidate positions $\left\{P_{o1},P_{o2},P_{o3},\ldots \right\}$. Ultimately, the robotic arm will only need to grasp one instance to be placed at the workstation. Therefore, for each object, the predefined reference direction is offline in its coordinate system, with the pose-specified axis set to the Z-axis. In this paper, an angle threshold is set to retain the valid poses by calculating the magnitude of the angle between the pose-specified axis and the reference direction. Positions where the defined angle is within the set maximum angle difference are retained, and the positions outside the maximum angle difference are removed. In addition to this, reachability and reliability were also used to evaluate the grasping point gesture further. Reachability refers to the ability of the robot to perform a grasping operation successfully in a real-world scenario. Reliability is the gripping error, which is mainly affected by the estimation of the object’s position. Therefore, the higher the accuracy of the pose estimation, the higher its reliability. Considering the robustness of the system, in this paper, we also sort the retained multiple postures and set a sorting rule to select the top N candidate bit-postures to ensure the uncertainty and complex environmental changes that may be encountered during the grasping process.

### 2.6. Evaluation of Indicators

The commonly used metrics for 6D pose estimation primarily include ADD [41] and ADD-S metrics [10]. For asymmetric targets, The ADD metric primarily computes the mean pairwise distances between the target vertices and the points obtained through the predicted ground truth pose transformation.

The evaluation metric employed in this study is the average distance (ADD) methodology. Given the ground truth pose $\left[R\vee T\right]$ and predicted pose $\left[R^{\prime }\vee T^{\prime }\right]$, ADD computes the mean distance between the 3D model points transformed by the predicted pose $\left[R^{\prime }\vee T^{\prime }\right]$ and the ground truth pose $\left[R\vee T\right]$. The validity of the positional information is determined only when the ADD-S metric is less than 10% of the diameter of the 3D model.

(14) $$ADD=\frac{1}{m}\sum_{x\in M}\left\|\left(Rx+t\right)-\left(R^{\prime }x+t^{\prime }\right)\right\|$$

where x is the vertex in the 3D model M point cloud, m is the total number of points in the point cloud. The predicted positions are denoted as R and t. The variables $R^{\prime }$ and $t^{\prime }$ represent the real positional attitude information.

ADD-S [40] is a performance metric that quantifies the average distance between symmetric objects and their nearest neighbors. The ADD-S metric calculates the mean distance between the point obtained from the predicted pose transformation $\left[R^{\prime }\vee T^{\prime }\right]$ and the nearest point on the 3D model transformed by the ground truth pose $\left[R\vee T\right]$. The ADD-S metric calculates the average distance between each 3D point under the predicted pose $\left[R^{\prime }\vee T^{\prime }\right]$ transformation and the nearest point on the 3D model under the ground truth pose $\left[R\vee T\right]$ transformation. The calculation of the area under the ADD-S curve (AUC) [38] is performed in this study.

(15) $$ADD-S=\frac{1}{m}\sum_{x_{1}\in M}\underset{x_{2}\in M}{min}\left\|\left(Rx_{1}+t\right)-\left(R’x_{2}+t^{\prime }\right)\right\|$$

where $x_{1},x_{2}$ are the closest points between the two poses.

## 3. Cases

Related experiments are discussed in this section. It is divided into two main parts: (1) The AUC performance of ADD or ADD(-S) was assessed using the publicly available datasets LineMod [39] and YCB-Video [10]. (2) Establishing a real dataset and deploying the model on a real robot grasping platform for experiments on robot grasping of low-texture industrial parts in cluttered scenes.

### 3.1. Datasets

(1) LineMod dataset [39]. It includes 13 distinct low-textured object shapes, 3D models, and corresponding ground truth poses. The image encompasses the complete pose space surrounding the scene object, along with a multitude of 2D and 3D noise waves in close proximity and at varying distances, making the scenes more representative of real-world conditions. It is, therefore, suitable for 6D attitude estimation of low-textured objects in cluttered environments. This paper uses the same training and testing datasets to compare with previous works.

(2) YCB-Video dataset [10]. In this paper, we perform multi-object attitude estimation of the YCB-Video dataset and enhance the data on its benchmark to achieve better results. According to the study conducted in [17], the training and test sets have been established, which contain 21 objects and are heavily occluded, enabling multi-target attitude estimation.

(3) Real-world dataset. In this paper, a new real-world dataset for pose estimation of low-textured industrial objects is produced to train and evaluate the proposed model approach. In different indoor scenarios, a single image contains several metal workpieces that need to be machined. This paper also uses data augmentation in the dataset to support the network structure. The dataset encompasses various scenarios with 2000 images per scenario. The training set accounts for 70 percent of the data, while the remaining portion is allocated for testing purposes.

### 3.2. Training Details

The approach presented in this study is built upon the PyTorch [42] deep learning framework. In the bidirectional dense feature extraction phase, the codec module follows the network structure used in FFB6D [17]. The RGB branch of the entire network utilizes the pre-trained ResNet34 [43] as its encoder and employs PSPNet [44] as its decoder. The RandLA Net [36] architecture is employed as the underlying network framework for extracting features from point clouds. The $N_{P}\times N_{f}$ feature matrix obtained from the fusion of these two branch features serves as input for achieving subsequent semantic segmentation, instance segmentation, and attitude estimation performance of the target object. The network is trained using an end-to-end methodology, implementing a step-wise learning rate decay strategy and an adaptive matrix (Adam) optimizer. The parameter settings for training are presented in Table 1.

### 3.3. Robot Grasping Experiment

To substantiate the method’s validity, reconstructing an experimental platform for automated manipulation of industrial components. Using a KUKA robot and 3D industrial camera as the hardware platform, the recognition and pose estimation of the workpiece in a scattered and occluded scene and accurate grasping were done for testing.

**Figure 7:**
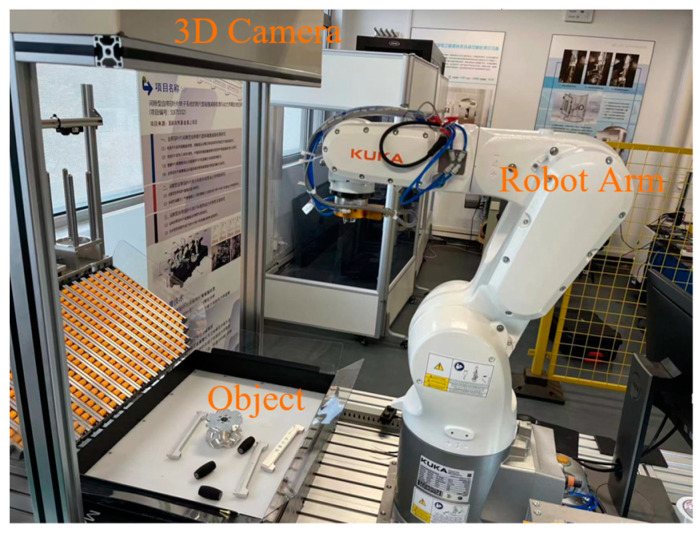
The experimental platform dominated by the 3D industrial camera, KUKA robotic arm, target object, and PLCS7-1200 is mainly used for industrial parts disordered gripping use.

**Figure 8:**
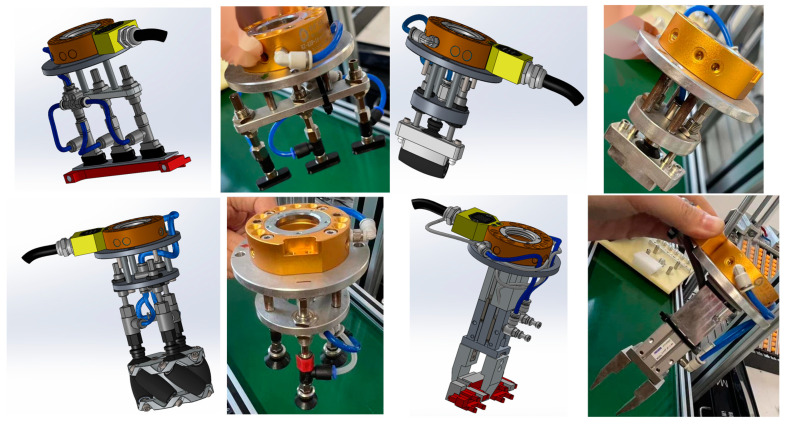
In the suction device, each part corresponds to one kind of suction device; the first row consists of two sets of fixtures: the beam fixture and the roller fixture. The second row includes the suction device for the wheel fixture and the base fixture; the left side of each set of fixtures is its 3D model, and the right side is the real fixture.

As shown in Figure 7, industrial cameras can acquire depth information of RGB images and scenes and convert the depth maps into point cloud maps using the camera’s internal reference. In order to avoid collision with other workpieces when the robot grasps the workpiece, this paper uses a suction cup to suction the workpieces, as shown in Figure 8.

**Figure 9:**
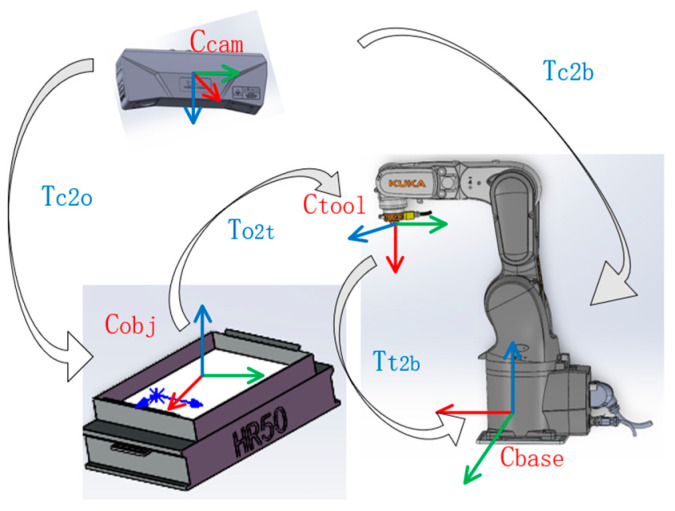
Coordinate transformation between the camera, robotic arm and object of the robotic gripping platform.

The coordinate frames of the industrial camera, workpiece, and robot are defined as shown in Figure 9. $T_{c2b}$, $T_{c2o}$ and $T_{t2b}$ denote the transformations between the coordinate systems of the camera, the object, and the robotic arms. The value of $T_{o2t}$ can be calculated from the previous three values $T_{c2b}$,$T_{c2o}$ and $T_{t2b}$. This results in robotic arm grasping.

**Figure 10:**
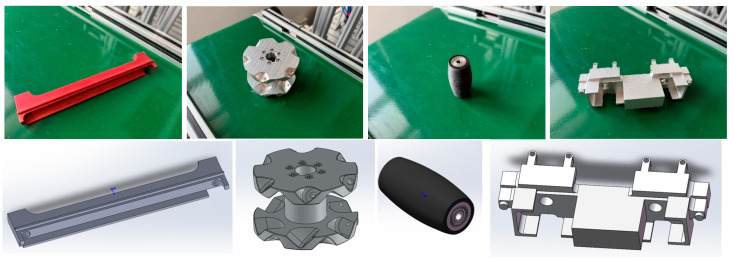
Four low-textured industrial parts. The first row shows, from left to right, the real object’s beam, hub, roller, and base. The second row shows the CAD models of the beam, hub, roller, and base from left to right.

**Figure 11:**
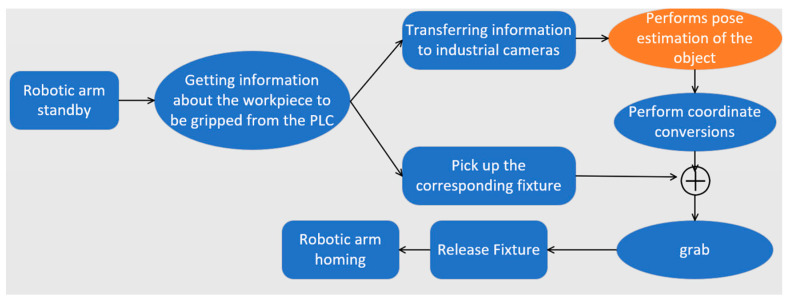
The robot arm first gets the signal from PLC to grasp the target workpiece, then sucks up the corresponding fixture, returns to the home position, waits for the gripping position information, and executes the gripping operation, and the whole process is shown above.

Figure 10 shows four different industrial parts used in this experiment: beam, hub, roller, and base. Gripping is considered successful if the KUKA robotic arm in the experiment is able to complete the gripping and suction within 5 s and successfully place the object in the tray without dropping it. Robotic gripping is a systematic task consisting of three key steps: reaching the object position by controlling the robotic arm, successfully suctioning the object, and then precisely lifting and accurately placing it. Therefore there are many factors that affect its successful grasping, such as the path planning of the robotic arm, the robot controller program, and our pose estimation algorithm for the target object. For path planning, as shown in Figure 11, the robotic arm will first suck up the corresponding suction cup when it gets the information about the target to be grasped and then return to the home position. After getting the 6D attitude information from the camera, based on the inverse kinematics, the robotic arm calculates the grasping position, then moves closer to the object until it reaches the final gripping position, then grips the object and lifts it, finally placing it on the pallet next to it.

## 4. Results and Discussion

The pose information of an object is estimated using RGB and depth images as inputs to the network, and the method’s effectiveness is validated on LineMod [31], YCB-Video [10] datasets, and real datasets.

**Figure 12:**
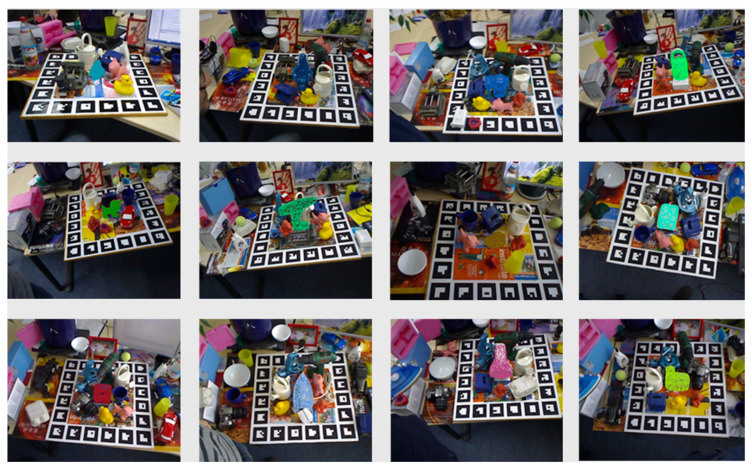
Pose estimation results on LineMod dataset.

**Figure 13:**
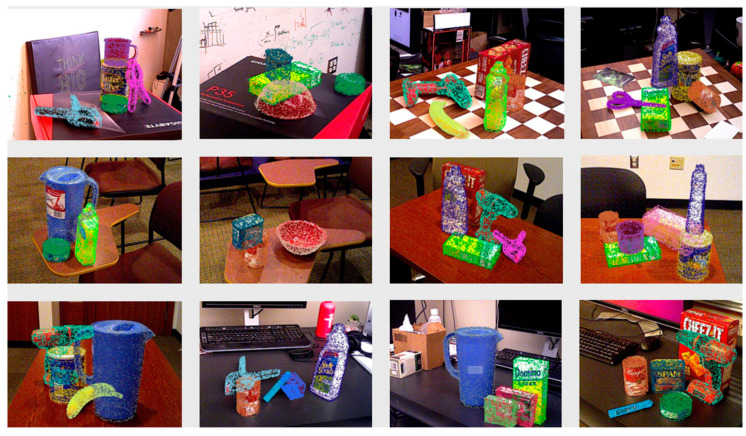
Pose estimation results on the YCB-Video dataset.

Figure 12 and Figure 13 show the pose estimation results in this paper on the Linemod [31] dataset and the YCB-Video [10] dataset. In this paper, the pose estimation of the proposed method for a single target is evaluated on the LineMod [31] dataset. The performance evaluation on the ADD-based LineMod [31] dataset is shown in Table 2. In this paper, the proposed modeling approach is evaluated on the LineMod [31] dataset against methods such as PointFusion [45] and DenseFusion [7]. Experiments show that the method is 26% more accurate in pose estimation than PointFusion [45], 5.4% higher than DenseFusion [7], and 0.7% higher than G2L-NET [46]. In addition to this, this research also performs performance evaluation on ADD(-S)-based YCB-Video dataset: as shown in Table 3, this method score is 20.5% higher than the results of RGB-based PVNet [32], and 2.1% and 1.2% higher than the RGBD-based PVN3D [8] and FFB6D [17], respectively. Compared to other state-of-the-art methods, the method proposed in this paper performs well on LineMod [31] and YVB-Video [10] datasets.

**Figure 14:**
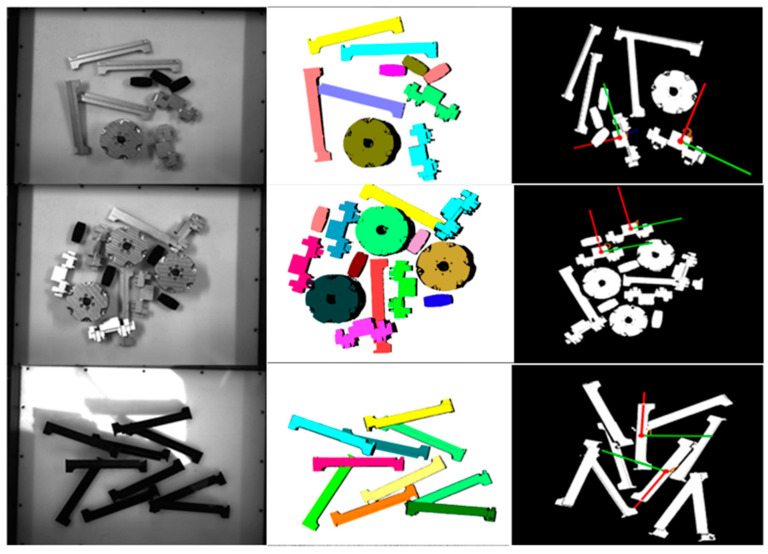
Pose estimation results on a real dataset. The results after instance segmentation clustering is shown in the second column, and the third column shows the attitude estimation results of the target object.

Evaluation on real datasets. Experimental validation of the prediction ability of the attitude estimation network in real scenarios. The data presented in Figure 14 demonstrates that the model is very accurate for scene instance segmentation and pose prediction. Among the multiple pose information correctly estimated, the robotic arm eventually selects the object with the optimal pose for grasping and re-estimates the pose of the remaining objects in the scene.

**Figure 15:**
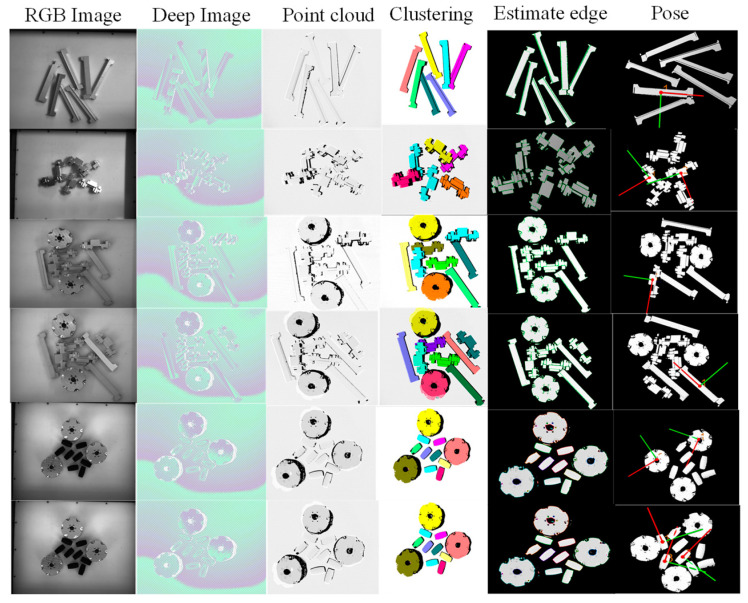
Pose estimation results obtained from testing in cluttered and occluded scenes. Each of these columns gives the RGB image, depth map, depth map converted point cloud, example point cloud clustering, edge estimation, and pose estimation results. This is a figure; schemes follow the same formatting.

**Figure 16:**
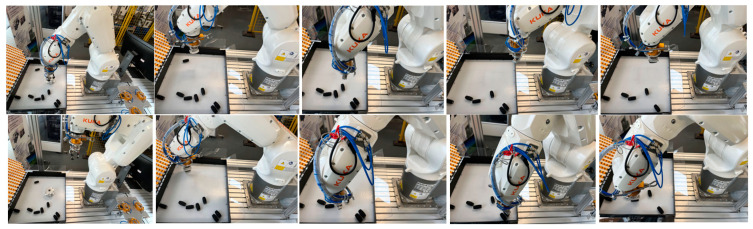

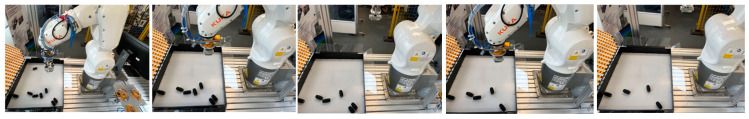
Some results of the robotic arm gripping experiments are performed in the real scene. Two kinds of workpieces are selected as gripping objects: a hub and eight wheels. Each column shows the process of converting the positional results obtained with the algorithm into positional information in robotic arm coordinates to make the robotic arm grasp each object.

As shown in Figure 15, robot grasping experiments were conducted for real clutter scenarios covering different combinations of single industrial parts and multiple industrial parts. Each column shows the RGB image, the depth map, the point cloud obtained from the depth map conversion, the example point cloud clustering results, the edge estimation, and the pose estimation results. After the instances are segmented, the point cloud alignment is performed by means of appearance edge matching, and the optimal one-candidate pose is found based on the confidence level calculated from the angle thresholds set for the target instance’s normal direction and the reference direction. To prevent encountering uncertain environmental changes, this paper retains the top 2 ranked postures, and finally, the robotic arm executes optimal grasping. As shown in Figure 16, after an object is selected, the scene is re-photographed to obtain a point cloud for the next object to be grabbed. This experiment further validates the model and the effectiveness of this grasping planning in robots picking up objects.

## 5. Conclusions

The study proposes the 3D point cloud pose estimation method based on geometric information prediction for robotic grasping applications. The issue was examined through appearance features and the geometric information of three-dimensional point clouds. The method improves the accuracy and processing speed of target object pose estimation. By incorporating edge appearance matching and leveraging key point features from point cloud data, this method significantly reduces computation time compared to traditional approaches like PPF, which requires traversing all point clouds for pose estimation. The proposed approach enhances the automation of robotic grasping tasks in industrial applications, improving speed, accuracy, and system reliability. We incorporate cross-entropy loss and discriminant loss functions into the instance network for untextured industrial objects in cluttered and occluded scenes. This fusion of semantic information with instance features allows for precise instance segmentation, accurately identifying object poses despite occlusion or clutter. Compared to the state-of-the-art methods, the pose estimation accuracy of the method proposed in this paper on untextured industrial objects performs 99.4% on the LineMod dataset and 93.9% on the YCB-Video dataset, which allows the robot to grasp objects in cluttered and occluded environments. These results highlight the method’s effectiveness in cluttered, occluded, and unstructured environments. Furthermore, the implementation of a robotic arm-gripping platform demonstrates the method’s real-world applicability. Future work will focus on extending the approach to handle unknown objects in cluttered scenes without the need for pre-existing 3D models, which could further broaden the scope of robotic grasping in dynamic environments.

## Figures and Tables

**Table 1:** Parameter settings for network training.

Parameters	Setting
Learning rate	0.0001 [11]
epoch	25 [11]
Batch size	3 [11]

**Table 2:** ADD-0.1d based performance evaluation of LineMod dataset.

Input	RGB		RGBD			
Method	PVNet [32]	DPOD [47]	PointFusion [45]	DenseFusion [7]	G2L-NET [46]	Ours
ape	43.6	87.7	70.4	92.3	96.8	98.6
benchvise	99.9	98.5	80.7	93.2	96.1	99.4
camera	86.9	96.1	60.8	94.4	98.2	99.5
can	95.5	99.7	61.1	93.1	98.0	98.4
cat	79.3	94.7	79.1	96.5	99.2	99.9
driller	96.4	98.8	47.3	87.0	99.8	100.0
duck	52.6	86.3	63.0	92.3	97.7	98.4
eggbox	99.2	99.9	99.9	99.8	100.0	99.9
glue	95.7	96.8	99.3	100.0	100.0	99.8
hole-puncher	82.0	86.9	71.8	92.1	99.0	99.8
iron	98.9	100.0	83.2	97.0	99.3	99.7
lamp	99.3	96.8	62.3	95.3	99.5	99.5
phone	92.4	94.7	78.8	92.8	98.9	99.7
Average	86.3	95.2	73.7	94.3	98.7	99.4

**Table 3:** ADD(-S) evaluation on the YCB-Video dataset.

Input	RGB		RGBD			
Method	PVNet [32]	PoseCNN [10]	DenseFusion [7]	PVN3D [8]	FFB6D [17]	Ours
	ADD(S)	ADD(S)	ADD(S)	ADD(S)	ADD(S)	ADD(S)
002 master chef can	81.6	82.1	70.7	80.5	80.6	80.8
003 cracker box	80.5	76.5	86.9	84.8	94.6	95.1
004 sugar box	84.9	83.9	90.8	96.3	96.6	96.8
005 tomato soup can	78.288.3	81.0	84.7	88.5	89.6	88.1
006 mustard bottle		90.2	90.9	96.2	97.0	97.5
007 tuna fish can	62.2	87.9	79.6	89.3	88.9	91.3
008 pudding box	85.2	79.0	89.3	95.7	94.6	93.2
009 gelatin box	88.7	87.2	95.8	96.1	96.9	95.8
010 potted meat can	65.1	78.7	79.6	88.6	88.1	89.6
011 banana	51.8	86.0	76.7	93.7	94.9	94.5
019 pitcher base	91.2	76.9	87.1	96.5	96.9	97.0
021 bleach cleanser	74.8	71.5	87.5	93.2	94.8	93.8
024 bowl	89.0	69.5	86.0	90.2	96.3	95.7
025 mug	81.5	78.1	83.8	95.4	94.2	95.2
035 power drill	83.4	72.5	83.7	95.1	95.9	96.3
036 wood block	71.5	65.4	89.5	90.4	92.6	92.8
037 scissors	54.8	56.9	77.4	92.7	95.7	97.2
040 large marker	35.8	71.5	89.1	91.8	89.1	89.9
051 large clamp	66.3	50.1	71.5	93.6	96.8	96.9
052 extra-large clamp	53.9	45.3	70.2	88.4	96.0	95.8
061 foam brick	80.6	87.9	92.2	96.8	97.3	97.6
Average	73.4	75.1	82.9	91.8	92.7	93.9

## Data Availability

The data presented in this study are available on request from the corresponding author due to laboratory research privacy policy.
